# Robotic Rehabilitation and Multimodal Instrumented Assessment of Post-stroke Elbow Motor Functions—A Randomized Controlled Trial Protocol

**DOI:** 10.3389/fneur.2020.587293

**Published:** 2020-10-22

**Authors:** Alessandro Pilla, Emilio Trigili, Zach McKinney, Chiara Fanciullacci, Chiara Malasoma, Federico Posteraro, Simona Crea, Nicola Vitiello

**Affiliations:** ^1^The BioRobotics Institute, Scuola Superiore Sant'Anna, Pontedera, Italy; ^2^IRCCS Fondazione Don Carlo Gnocchi, Firenze, Italy; ^3^Rehabilitation Department, Versilia Hospital, USL Nord Ovest Toscana (AUSLTNO), Lido di Camaiore (LU), Italy; ^4^Department of Excellence in Robotics & AI, Scuola Superiore Sant'Anna, Piazza Martiri della Libertà, Pisa, Italy

**Keywords:** stroke, robotic rehabilitation, instrumented spasticity assessment, exoskeleton, upper limb, joint stiffness, functional rehabilitation

## Abstract

**Background:** The reliable assessment, attribution, and alleviation of upper-limb joint stiffness are essential clinical objectives in the early rehabilitation from stroke and other neurological disorders, to prevent the progression of neuromuscular pathology and enable proactive physiotherapy toward functional recovery. However, the current clinical evaluation and treatment of this stiffness (and underlying muscle spasticity) are severely limited by their dependence on subjective evaluation and manual limb mobilization, thus rendering the evaluation imprecise and the treatment insufficiently tailored to the specific pathologies and residual capabilities of individual patients.

**Methods:** To address these needs, the proposed clinical trial will employ the NEUROExos Elbow Module (NEEM), an active robotic exoskeleton, for the passive mobilization and active training of elbow flexion and extension in 60 sub-acute and chronic stroke patients with motor impairments (hemiparesis and/or spasticity) of the right arm. The study protocol is a randomized controlled trial consisting of a 4-week functional rehabilitation program, with both clinical and robotically instrumented assessments to be conducted at baseline and post-treatment. The primary outcome measures will be a set of standard clinical scales for upper limb spasticity and motor function assessment, including the Modified Ashworth Scale and Fugl-Meyer Index, to confirm the safety and evaluate the efficacy of robotic rehabilitation in reducing elbow stiffness and improving function. Secondary outcomes will include biomechanical, muscular activity, and motor performance parameters extracted from instrumented assessments using the NEEM along with synchronous EMG recordings.

**Conclusions:** This randomized controlled trial aims to validate an innovative instrumented methodology for clinical spasticity assessment and functional rehabilitation, relying on the precision and accuracy of an elbow exoskeleton combined with EMG recordings and the expertise of a physiotherapist, thus complementing and maximizing the benefits of both practices.

**Clinical Trial Registration:** www.ClinicalTrials.gov, identifier NCT04484571.

## Introduction

Stroke is an acute cerebrovascular injury, resulting in numerous cognitive, sensory, and motor deficits among survivors, including complete or partial hemiparesis, loss of strength and dexterity, and/or severe limb rigidity, thus impairing activities of daily living (ADL) and promoting further pathological progression (1, 2). A major contributor to this symptom progression is muscular *spasticity* (3, 4), first defined by Lance (5) as a velocity-dependent hyperactivity of the tonic stretch reflex, causing hyper-resistance to muscle elongation (6). Accordingly, spasticity alleviation has been a primary clinical aim of both pharmacological [e.g., via botulinum toxin (4)] and physical therapies for stroke rehabilitation (7, 8). For the upper limb, robotically assisted training (RAT) has been demonstrated to mitigate spasticity and improve function (9–12), but additional validation remains necessary for incorporation of RAT into official clinical guidelines for post-stroke spasticity (13).

However, the phenomenon of *spasticity* has been inconsistently defined and evaluated in clinical practice to date (14). While several clinical spasticity assessment scales are available (15), none are recommended or used by established consensus (14, 16). On this topic, a recent neurological consensus (14) emphasized that the term “spasticity” should be reserved exclusively for *velocity-dependent stretch hyperreflexia*, distinct from both general hypertonia (involuntary background activation) and mechanical tissue properties. However, current clinical assessment techniques encompass both neurological and non-neurological components in a single measure (6), as true of the most commonly adopted clinical spasticity assessment scale, the Modified Ashworth Scale (MAS). Further, its rater-dependent subjectivity and ordinal quantization render the MAS imprecise, unreliable, and of limited value for the clinical assessment and rehabilitation of post-stroke patients (17–21).

Accordingly, there exists a strong clinical need both for more effective treatments and for more precise and reliable instrumented evaluation methods in characterizing post-stroke joint stiffness, spasticity, and overall motor function, including both biomechanical and neuromuscular properties (14, 22). To this end, numerous robotically instrumented measures have been developed and investigated to evaluate upper-limb spasticity and motor function (22–25) and have demonstrated strong correlation with existing clinical scales (26). Such instrumented measures provide a better understanding of patient pathophysiology, enabling spasticity treatment to be integrated within functional rehabilitation programs specifically designed and adapted to individual patient needs (13, 21). To this end, exoskeletal robotics (27, 28) offer the unique capability both to precisely characterize and assist movements at the level of individual joints.

Based on a previous clinical study demonstrating basic device safety and feasibility in preventing typical increases of post-stroke spasticity (11), the current study protocol describes a randomized controlled trial (RCT) of the NEUROExos Elbow Module (NEEM) (10, 29), a powered elbow exoskeleton, for the treatment and characterization of post-stroke elbow spasticity and motor impairment.

## Materials and Methods

### Objectives

The primary study objective is to rigorously evaluate the efficacy of robotically assisted training as a novel modality for clinical rehabilitation of post-stroke elbow spasticity and motor function, in a randomized controlled trial relative to conventional physical therapy. Secondarily, the study aims to validate a novel instrumented method for elbow stiffness/spasticity evaluation, by combining biomechanical (robotically recorded) and electromyographic (EMG) parameters.

### Study Protocol Design

The present clinical study protocol describes a single-blind randomized controlled trial comparing 4 weeks of RAT using the NEEM robotic elbow exoskeleton to a control group receiving a matched volume of conventional physiotherapy. The primary outcome measures are the Modified Ashworth Scale (MAS) and the upper-extremity Fugl-Meyer Assessment (FMA) scale, two standard clinical scales used to assess upper-limb spasticity and functionality in neurological patients. Subjects are randomly assigned either to the control group (CG), or to the treatment (robotic) group (TG). Both groups will receive a conventional 4-week upper-limb physiotherapy program, consisting of repeated elbow flexion/extension movements: control group patients will focus on manual mobilizations of the limb while aided by the therapist, while the robotic group will receive the same net treatment volume via the robotic device. The treatment group will be further divided into two levels (sub-groups), based on pathological severity: Level 1 corresponds to an FMA score equal or below 28, and Level 2 with FMA >28. The two sub-groups will receive different combinations of robotically assisted *passive* and *active* mobilization treatment modalities, according to their differing clinical needs. Level 1 therapy is designed for patients with very low residual mobility, focusing on *passive mobilization* of the affected limb using a *robot-in-charge* control paradigm (29), where the robot moves the user's arm through a pre-defined spatio-temporal trajectory without requiring his/her active contribution, so as to entrain desired movement patterns and/or reduce joint stiffness (including spasticity). In addition to passive mobilization, Level 1 participants will also perform a limited number of *active mobilization* exercises, using a *patient-in-charge* paradigm (29) in which the user initiates and follows defined movement trajectories, receiving robotic assistance only *as-needed*. According to their higher motor function at baseline, Level 2 subjects will perform a greater proportion of active mobilizations relative to passive exercises (3:1), to emphasize the development of upper limb strength and motor control. Subjects in both treatment sub-groups will also be evaluated for their voluntary movement capacity in each session, via a brief bout of active movements performed with the robotic device in *transparent* (unassisted) mode.

### Participants and Recruitment

All procedures conform to the Declaration of Helsinki and have been approved by the Ethical Committee for the Azienda USL Toscana Nord Ovest (AUSLTNO) and the Italian Ministry of Health.

The study is conducted at the Recovery and Functional Rehabilitation department of Ospedale Versilia (Lido di Camaiore, Italy), in collaboration with The BioRobotics Institute of Scuola Superiore Sant'Anna. It involves 60 post-stroke patients with right-sided hemiplegia, randomly allocated either to the control (*n* = 30) or treatment (*n* = 30) group. The sample size of each group was calculated based on the hypothesis of a post-treatment MAS score reduction of 0.5 points (compared to pre-treatment, from an average of 2.5 ± 0.70 SD at baseline) with an alpha value of 0.05 and power equal to 80%. (30).

According to inclusion criteria, patients should: (1) be in the age range 18–79; (2) have sustained unilateral hemorrhagic or cerebro-vascular ischemic event in sub-acute (< 6 months) or chronic (> 6 months) phase; (3) experience a functional motor deficit of the right arm (ranging from mild to severe hemiplegia or paresis); (4) have residual cognitive capabilities sufficient for the understanding of basic instructions (Mini-Mental State Examination score > 24); (5) exhibit lack of pain upon passive mobilization of the limb; (6) be able to sign an informed consent. On the contrary, patients are excluded from the study in case of: (1) unstable state of general health; (2) inability to remain in a seated position for a prolonged period (1 h or more); (3) use of a cardiac pacemaker or other active implanted medical devices; (4) passive range of motion (ROM) lower than 10 degrees. In addition, patients who received botulinum toxin injections within 3 months prior to the participation to the study or individuals who follow any additional pharmacological treatment that may alter the outcome of the study are excluded from the participation to the study. Sudden changes in subjects' health conditions, severe alterations in their psychophysical well-being or the inability to attend more than three daily consecutive sessions will lead to the exclusion from the study.

Patients are recruited from across Tuscany via referral to Versilia Hospital. Once eligibility is confirmed, patients sign an informed consent and are invited for a traditional assessment session with a therapist blinded to their group assignment. Afterwards, patients are randomly allocated to either arm via a random number generator algorithm in blocks of ten. Patients perform daily rehabilitation therapy (5 sessions per week) for 30 min over a 4-week period, for a total of 20 treatment sessions. Subjects assigned to the control group perform 30 min of conventional therapy exercises; patients assigned to the treatment group are subject to 30 min of treatment with the elbow exoskeleton.

**Figure 1:**
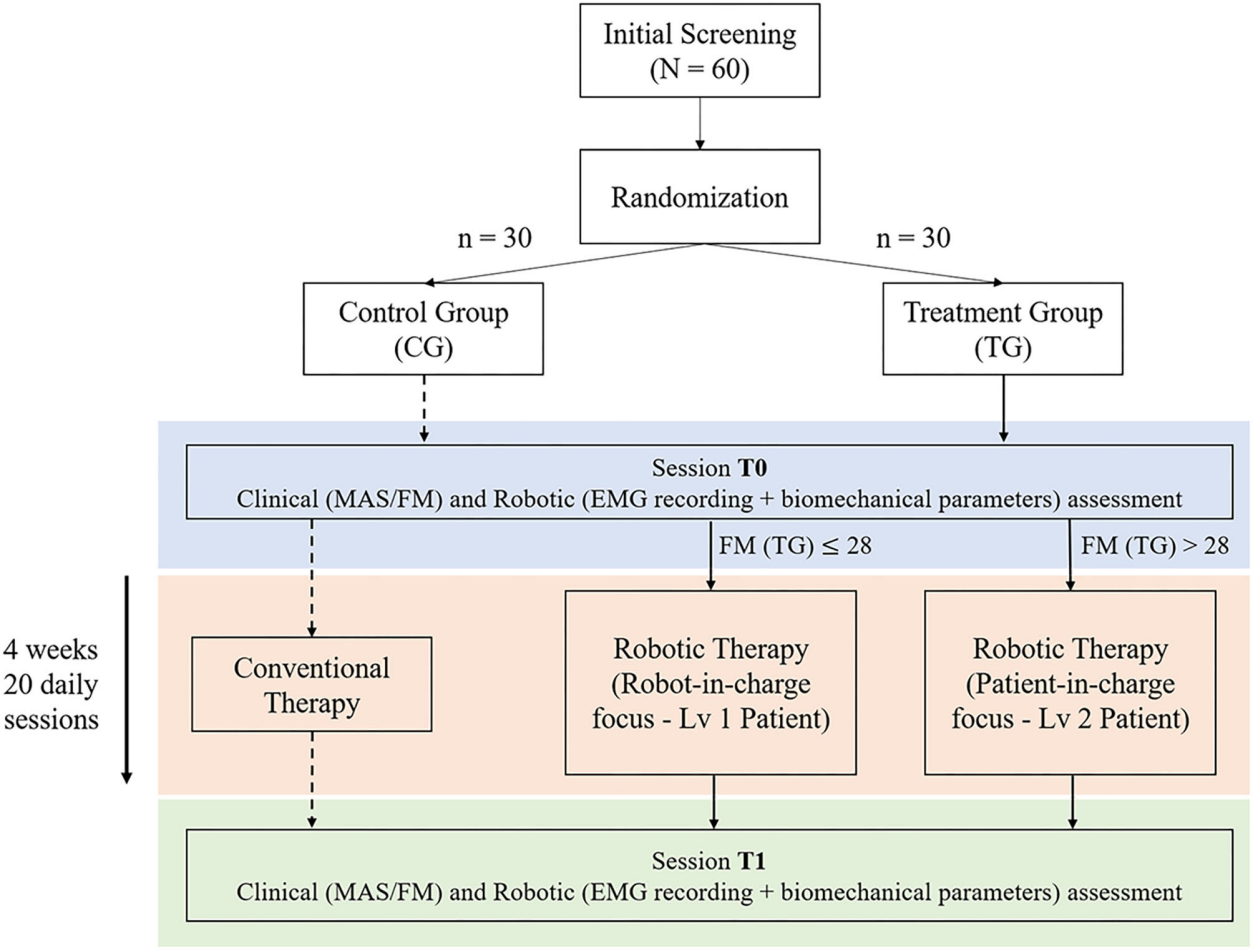
Protocol overview—Treatment Group (TG) patients are divided in two exercise levels based on the upper Fugl-Meyer Assessment (FMA) score. Both groups conduct an initial assessment (T0) followed by 4 weeks−20 days treatment therapy, concluding with an additional assessment session (T1).

Subjects in both groups will undergo both clinical and instrumented evaluations (using the NEEM device) at two time points: pre-treatment (baseline, session T0) and post-treatment (session T1) within 1 week after the end of treatment. Each clinical assessment will include the evaluation of MAS and FMA scores, assessed in blinded fashion. All robotic assessments will include the evaluation of the elbow ROM, as well as other biomechanical and electromyography parameters extracted under different dynamic conditions. Figure 1 represents a scheme of the experimental protocol.

### Description of the Robotic Platform

**Figure 2:**
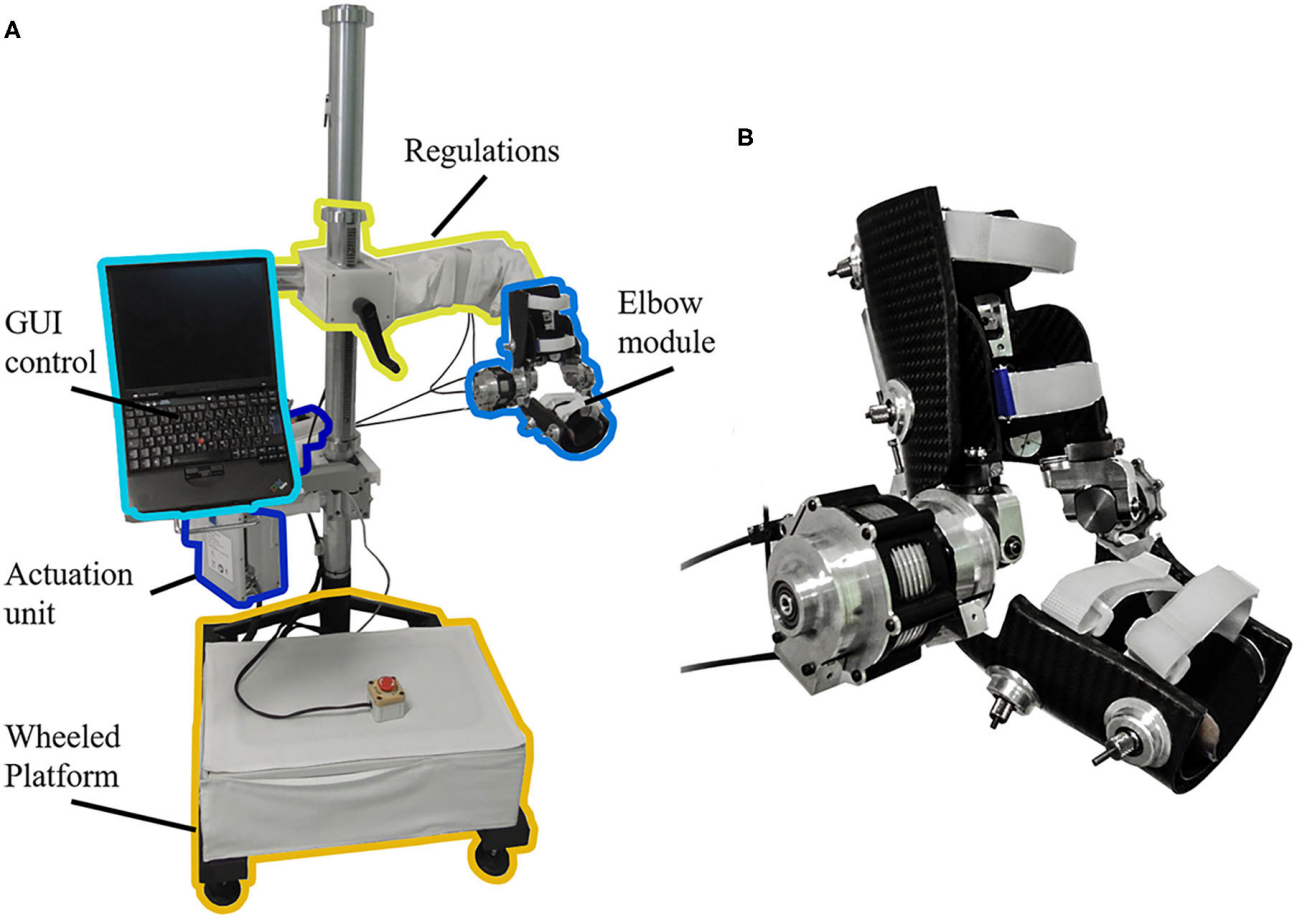
**(A)** NEUROExos Elbow Module platform, including: passive regulations to adjust the height and orientation for each patient; actuation unit; wheeled platform to move the device in the 3-D workspace; graphical user interface (GUI) control via a laptop. **(B)** Detail of the elbow module.

The NEEM is a highly ergonomic powered exoskeleton for elbow mobilization and neurorehabilitation, designed and developed at The BioRobotics Institute (Scuola Superiore Sant'Anna, Pisa), and licensed to its spin-off company IUVO Srl (Pontedera, Italy). In addition to its powered actuation of elbow flexion/extension (F/E) movements, the NEEM includes a 4-degrees-of-freedom (DoF) self-aligning mechanism to accommodate for the laxity of the human elbow joint (31), a height regulation mechanism, and a spherical joint for the positioning of the robot in the workspace (Figure 2).

The system offers two working modalities: (1) *Joint position control* mode (“robot-in-charge”), where the robot moves the user's arm through a pre-defined trajectory; (2) *Joint torque control* mode (“patient-in-charge”), in which the robot provides a commanded torque (either assistive or resistive) to the user's arm. When the desired torque is zero, the device works in *transparent mode*, allowing free movement by the user. The NEEM can provide synchronous joint angle-torque recordings in any of its modes.

**Figure 3:**
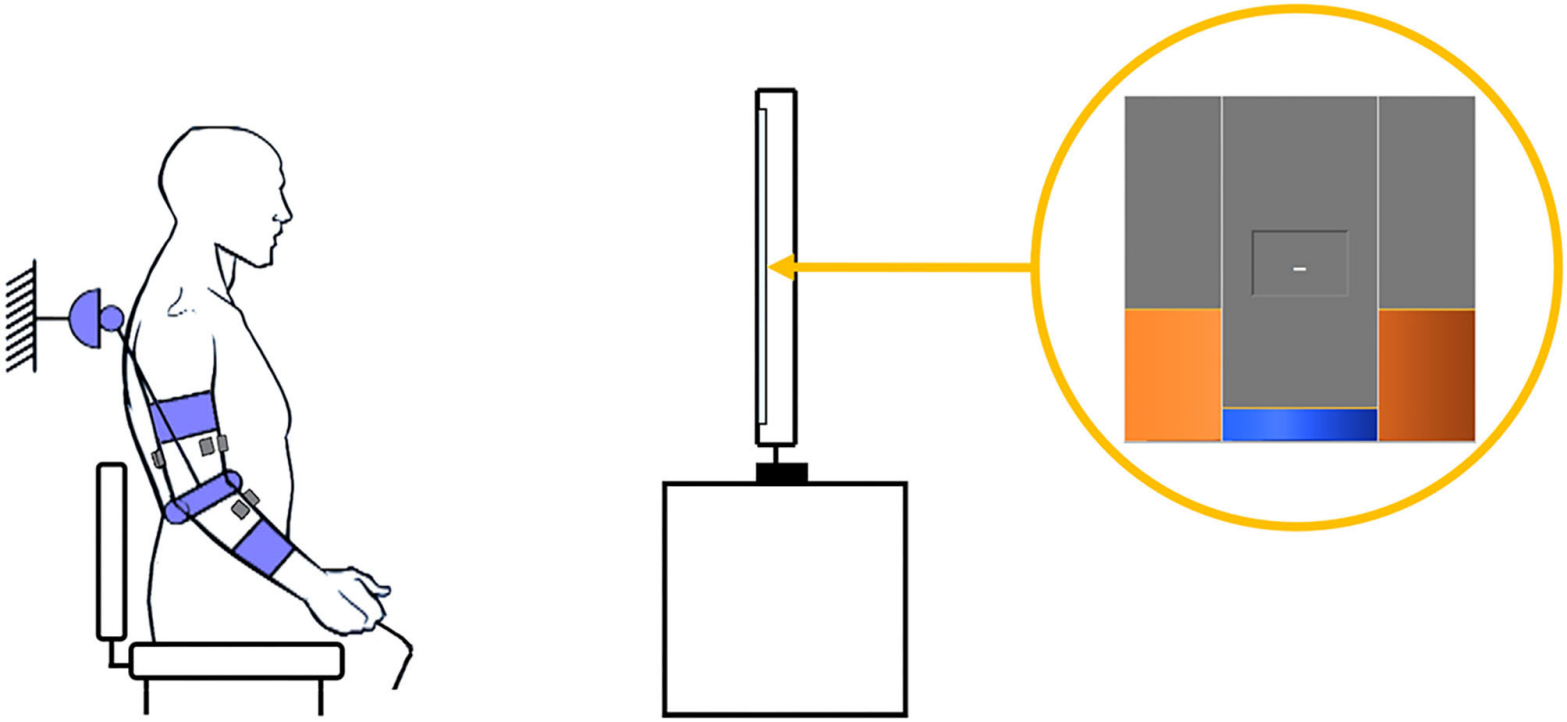
Example of an experimental setup. The patient, donning the exoskeleton and with electrodes positioned on the impaired arm, follows the reference angle (orange) depicted on the screen while keeping the actual joint angle (blue) as close as possible.

The present study will use robot-in-charge mode to effect *passive mobilization* of the subject's arm (with no voluntary user input), and patient-in-charge mode (either with the robot providing assistive torque or in the *transparent mode*) for *active mobilization* exercises. Both passive and active ROM (pROM, aROM) are recorded in transparent mode: pROM with the limb manually mobilized by the therapist, and aROM via voluntary movements performed by the subject. The NEEM provides a graphical user interface (GUI) that allows the experimenter to configure the assessment/rehabilitation protocol, according to the therapist's guidance and patient's needs. Exercise instructions and target joint trajectories are displayed to the patient in a dedicated GUI module, appearing on a separate screen (Figure 3).

The NEEM system can be integrated with an external EMG recording device via a synchronization signal (square wave) routed through a dedicated wired channel, with a maximum delay (uncertainty) of 10 ms. In this study, three EMG channels will be used in the robotic assessment to record muscle activity from the *biceps brachii* (BB), *triceps brachii* (TB), and *brachioradialis* (BR) at a sampling frequency of 1 kHz.

### Experimental Protocol

The protocol is divided into 2 assessment sessions and 20 treatment sessions for both arms of the study, for a total of 22 sessions structured in exercise blocks focused on elbow mobilization.

**Figure 4:**
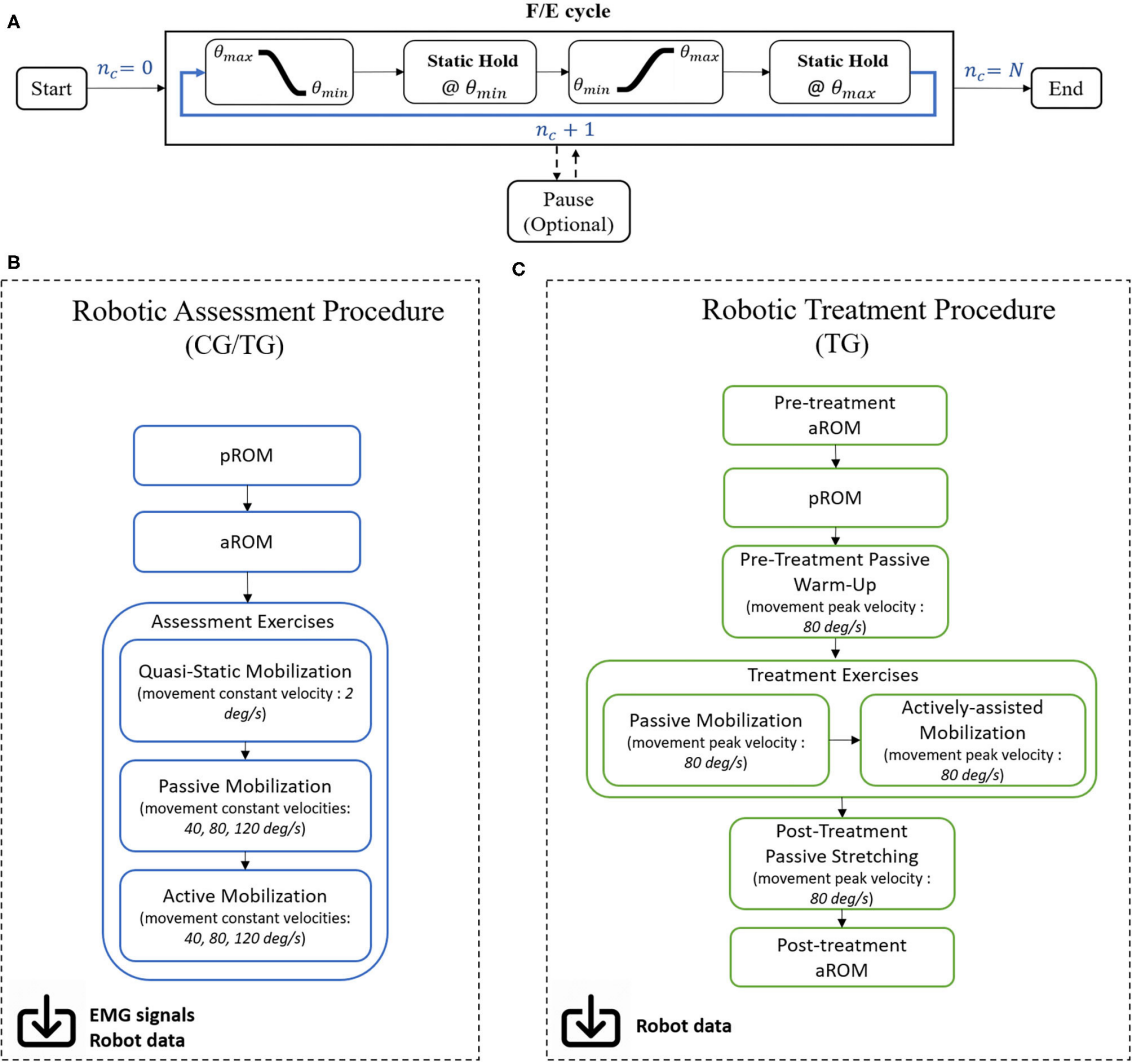
**(A)** Each exercise block consists of ramp/sigmoidal movements from the maximum ROM angle to the minimum, followed by a Static Hold time interval where the patient is asked to maintain the given position. Movement is then inverted and repeated with the same modality until the number of performed cycles **n**_**c**_ equals the desired number of movements *N*. **(B)** Workflow for the robotic assessment and **(C)** treatment procedures.

A robotic mobilization exercise (Figure 4A) includes *N* repeated F/E elbow movements from the maximum angle (θ_max_ - flexed elbow) to the minimum angle (θ_min_ - extended elbow) and vice versa, at a set peak velocity. Between movements, the subject is asked to hold the current angular position (*Static Hold* state), lasting a set time interval of 3 or 5 s, respectively for treatment and assessment exercises. The exercise ends when the number of performed F/E movements *n*_*c*_ reaches the desired value *N*. An optional *Pause* state is present to better suit the performance to the subject's needs: in this case, the system switches to a *transparent* modality, in which the user is free to move and relax; as soon as the subject is ready, the exercise can start anew from the last performed F/E cycle *n*_*c*_.

Exercises are implemented in the form of consecutive training blocks in assessment and treatment sessions, hereby detailed (Figures 4B,C).

### Assessment Procedure

The robotic assessment procedure (Figure 4B) is conducted on the first and within 1 week after the last days of the treatment period. Upon arrival, subjects are informed about the study and the procedures, and sign the consent form. Then they are prepared for the application of the EMG electrodes following the SENIAM guidelines (32). Commercial Ag/AgCl electrodes are placed on the BB, TB and BR muscles, and fixed in place by using tape and sterile dressing. Before fixing the electrodes, visual inspection of the signal is conducted to verify the quality of the signal and that the baseline noise is sufficiently low. Then, the patient dons the exoskeleton with the help of the therapist and the experimenter, and the robot configuration is adjusted to achieve proper fitting with the subject in a comfortable, seated, upright position, with the shoulder in slight flexion (10–30°), slight abduction (10–30°), and internal/external rotation so as to render F/E movements in the approximate sagittal plane.

The assessment procedure starts with the measure of the patient's passive range of motion (pROM). With the device in transparent mode, the therapist moves the affected limb to the maximum and minimum angular positions reached without experiencing resistance to the movement or elicit pain. The difference between the maximum and minimum angular values recorded within three consecutive flexion-extension movements are stored as pROM. An active range of motion (aROM) is then recorded in a similar fashion, during active movements performed by the patient with the device in transparent mode. Elbow mobilization is then performed, consisting of three consecutive exercises: (1) passive quasi-static, (2) passive, and (3) active (transparent mode). The order of the exercises is fixed.

The passive quasi-static exercise includes 5 F/E constant-velocity movements within the pre-recorded pROM, during which the patient is asked to relax as the system mobilizes the limb at 2 deg/s: the main purpose of this exercise is to evaluate muscle and soft tissue resistance to the imposed stretch, by minimizing the contribution of the robot's dynamic and inertial components, which are negligible at constant low speeds.

The passive and active assessment exercises consist of 36 constant-velocity F/E cycles executed at three different velocities (12 cycles per velocity): 40 deg/s, 80 deg/s, 120 deg/s. The velocity order is fixed, and it has been chosen to investigate the occurrence of velocity-dependent spastic contractions of arm muscles. During passive exercises, the patient is asked to relax while the device moves the hindered limb within the pROM. Active exercises, instead, require a dynamic involvement by the patient, who is asked to follow a reference angle on the GUI, moving within the aROM using fully available motor skills.

### Treatment Procedure

The treatment protocol (Figure 4C) lasts for 4-weeks, for a total of 20 sessions. At the beginning of each robotic treatment session, the subject is seated in front of a screen and is helped in donning the exoskeleton with the aid of the therapist and experimenter. The position of the patient with respect to the exoskeleton, as well as the exoskeleton regulations are adjusted to replicate the same configuration as the initial assessment procedure. This will ensure that any variation of biomechanical parameters is not attributed to a different spatial configuration of the patient with respect to the exoskeleton. Due to time restrictions (each session must be conducted within 30 min), treatment sessions do not include EMG recording to maximize the treatment volume.

The treatment procedure starts with the recording of the aROM while wearing the device in transparent modality. Then, a quick (about 2.5 min) passive mobilization is performed within the pROM recorded during the initial assessment procedure (T0), at a fixed velocity of 80 deg/s. Purpose of this mobilization is to have a continuous monitoring of the extracted passive biomechanical parameters through the whole treatment period, with the same conditions as the initial assessment.

The patient is then asked to relax, and a new pROM is recorded with the aid of the therapist. Afterwards, the affected limb is passively moved within this pROM by the exoskeleton via sigmoidal movements at 80 deg/s of peak velocity; the duration of the exercise is set according the training level assigned at the initial screening: 10 min for level 1 patients, 2.5 min for level 2 patients.

Next, the subject is asked to perform an active-assisted exercise in the pROM, by following a reference F/E trajectory in the GUI. The device provides an assistive torque proportional to the error between the reference value and the measured angular value, weighed via a proportional assistance constant *K*_*p*_. A performance index (PI) measure is extracted from each active mobilization block, tying the user's daily performance to the cumulative error in each F/E cycle. The robotic input parameters of passive range of motion (used for both passive mobilization and active exercises) and assistance constant *K*_*p*_are individually adapted to the patient in each session, based on the instrumented measurement of pROM at the start of each session and the PI from the previous two sessions. Specifically, *K*_*p*_ is adjusted (increased or decreased by a set percentage, or else left unmodified) so as to maintain the PI within a moderate range relative to healthy controls, thus representing a “training zone” of functional difficulty where the user remains able to substantially complete the exercises yet is sufficiently challenged to elicit a training effect. The duration of the active-assisted exercise is 2.5 min for level 1 patients, and 10 min for level 2 patients. Two sets of passive and active mobilizations are performed.

At the end of the treatment session, the patient is asked to perform another 2.5 min of passive mobilization within the pROM of the initial assessment, followed by the recording of the aROM without assistance by the device. This procedure is performed to enable evaluation of intra-session changes in movement capacity.

### Outcome Measures

For the primary objective of the study, patients will be clinically evaluated at the end of the treatment period with the MAS and upper FMA assessment scales, following the same procedure adopted for the initial screening. Score differences between treatment and control group will be statistically analyzed to draw conclusions on the validity and efficacy of conventional and robotic approaches to clinical treatment and assessment practices. These constitute the primary outcome measures of the study.

Regarding the secondary objective, a wide array of biomechanical- and neuromuscular activity-related features will be extracted from the NEEM torque-angle data and from the EMG signals synchronized with the exoskeleton data, respectively.

Biomechanical parameters extracted from the robot in quasi-static exercise include:

- Maximum Extension Torque: the maximum torque value recorded during a static hold phase at the maximum extension angle (10);
- Zero Torque Angle: the angular value corresponding to null torque exerted by the robot; in this configuration, flexor and extensor torques (free of gravity force contributions) are equal and opposite and the system is ideally not applying any force to hold the elbow in position (10);
- Joint Impedance: measure of the total limb resistance to muscle elongation, computed as the ratio between the difference in joint torque over the corresponding change in joint angular position (33, 34); for constant, slow-speed movements, the main contribution to the joint impedance can be identified in the joint stiffness (i.e., rigidity).
- EMG signals recorded during quasi-static exercises are used as reference for the background muscle activation, where no spastic contractions should be elicited.

Biomechanical parameters extracted from the robot in passive exercises include Maximum Extension Torque, Zero Torque Angle and Joint Impedance (in this case also including a velocity-dependent viscoelastic component). Additionally, the normalized Torque-Angle Integral is extracted: the integral of the Torque-Angle curve, normalized to the pROM, represents a measure of the expended energy by the robot to move the patient's arm. Higher values correspond to limb stiffening, as the exoskeleton delivers more power to mobilize the limb in the ROM.

For the active exercises, the main outcome measure is the movement smoothness: recently, several studies (35, 36) have approached the question of how to classify and quantify movement “smoothness.” Unequivocally, healthy subjects show faster, more continuous movements compared to patients affected by neural deficits (37).

In addition to biomechanical parameters, other features are extracted from EMG signals during passive and active exercises (10):

- Stretch Reflex Onset: extrapolated from the EMG envelope, it is defined as the first sustained burst of muscular activity above the baseline value for at least 200 ms. The threshold for muscle activation is defined as the *mean*+2^*^ *standard deviation* value of the filtered EMG signal in the 100 ms time window prior to the imposed stretch (38);
- EMG Burst Duration: percentage of the F/E movement time during which EMG activity is present (39);
- Position Threshold: defined as the joint angular value associated to the Stretch Reflex Onset (10, 38);
- Muscle Co-activation: defined as the percentage of movement time in which simultaneous activation of agonist and antagonist muscles (i.e., BB and TB) is observed.

EMG outcomes will be interpreted with biomechanical parameters, with the goal to provide a comprehensive description of the biomechanical and neurophysiological components of joint resistivity. Finally, robotic and clinical measures will be investigated to provide a more detailed clinical picture of each patient by enriching the information content of the assessment scales.

### Statistical Analysis

In addition to descriptive statistics characterizing the study population and the assessment of potential clinical or demographic differences between groups via Pearson's chi-squared test, this study will employ a paired one-way analysis of variance (ANOVA) of all primary outcome measures, with repeated measures taken from baseline (T0) and post-treatment (T1). This series of tests will be contingent on confirmation of the normality of all data distributions using the Shapiro-Wilk normality test. If data are found not to conform to the null hypothesis of Gaussian distribution, then the parametric Mann–Whitney *U*-test (also known as Wilcoxon rank sum test) will be used in place of ANOVA, in pair-wise fashion.

## Discussion

The current RCT builds on a previous clinical study by Crea et al. (11) that used the NEEM for treatment of post-stroke elbow spasticity in a 2-week (10-session) robotic rehabilitation program, which successfully prevented typical increases in post-stroke spasticity, as measured via MAS and instrumented biomechanical parameters. The present study protocol seeks to further validate the results of the initial single-arm study using a randomized controlled design with a larger sample size over a longer (4-week) treatment period, further incorporating neuromuscular (EMG) activity recordings, new exercise modalities, refined biomechanical parameters, and a personally adaptive study design.

The proposed protocol extends both the previous work and the existing body of clinical literature on robotic upper limb stroke rehabilitation in several important ways. These contributions may be best understood within the framework offered by Duret et al. (40), whose review posited that effective upper limb stroke rehabilitation programs should employ repetitive, intensive, adaptive, and quantifiable therapy. These criteria are echoed in the American Stroke Association's Guidelines on Stroke Rehabilitation (13) and are all fulfilled by the current RCT, with specific focus on the treatment and assessment of elbow spasticity.

To achieve *repetitiveness*, our protocol employs joint trajectories that are first precisely repeated by the robot in a passive mobilization exercise, and then employed as target trajectories for the user to actively follow, with assistance provided *as-needed*. In this way, the delivered highly *intensive* physiotherapy is characterized by more repetitions of higher quality than can be achieved in traditional, manually assisted therapy alone. Indeed, a majority of positive effects in robotic rehabilitation studies to date have been attributed precisely to their ability to administer repetitive, intensive physiotherapy, regardless of modality (13).

For instance, in a randomized pilot study (*n* = 30), Fazekas et al. (9) employed passive mobilization of the shoulder and elbow via two industrial robots in patients with upper limb hemiparesis due to stroke or mild traumatic brain injury, with results demonstrating significant clinical improvements in elbow MAS with combined robotic-plus-conventional therapy, but not conventional therapy alone. Similarly, Posteraro et al. (41) employed a planar end-effector robot (MIT-MANUS) in a 10-session rehabilitation protocol, finding statistically significant improvements in the shoulder MAS, elbow passive ROM, and Motor Status Score at both the shoulder and elbow using two separate treatment protocols focusing on different movement patterns. By contrast, the stretch reflex—evaluated indirectly via the robotic parameter of minimum jerk deviation as an indicator of spasticity—showed no statistically significant decrease for either treatment protocol.

The aspect of *adaptiveness* is among the core strengths of this RCT design, which adapts to the needs and abilities of each patient on various time scales. First, during our program's active exercises, each repetition is facilitated by a robotic *assist-as-needed* paradigm, with assistive force proportional to the error between target and measured limb trajectory, thus prompting and enabling the subject to exert maximum voluntary control of the paretic limb. At the session level, the ROM used for both passive mobilization and active exercises is adaptively defined, based on the patient's passive ROM measured at the start of each session, via manual mobilization by the therapist with robot in transparent mode. In this way, the exercise workspace is adapted in each session to maintain optimal session-to-session progress both in elbow mobilization (for spasticity and stiffness reduction) and motor control. In addition to ROM, another key adaptive parameter modified in each treatment session is the level of assistance (K_p_) for the active exercises, which is iteratively adjusted based on the Performance Index of the most recent sessions, so as to maintain performance within a moderate range, hypothesized to approximate an “optimal challenge point” for motor recovery. Indeed, it has been noted in numerous studies that patient engagement, voluntary participation, and bounded variability in robot-assisted exercises are key components of successful upper and lower limb rehabilitation (42–45), and our assist-as-needed assistance accomplishes all three. Finally, at the protocol level, the selection of a robotic rehabilitation program based on the two-tiered functional classification of subjects according to their clinical assessments (FMA score) aims to provide the balance of treatment modalities (passive vs. active mobilization) best suited to each patient's needs.

The proposed RCT design excels beyond previous robotic rehabilitation studies and the current state of the art also in the provision of precise, *quantitative* measures, via comprehensive clinical and instrumented assessments at the start and finish of our custom 4-week rehabilitation program. Significantly, our protocol involves a thorough neuro-mechanical characterization, comprising of synchronous biomechanical and neurophysiological (EMG-based) parameters in response to passive stretch under both quasi-static and dynamic conditions at varying velocities, as well as during unassisted active movements. Critically, the NEEM's series-elastic actuator (SEA) architecture ensures patient safety while allowing more precise joint angle-characterization than achievable with traditional robotic systems. Together, this set of instrumented measures enables the quantitative distinction between neural and non-neural components of elbow hyper-resistance, as well as the isolation of velocity-dependent spasticity, as recommended by the van den Noort consensus (14). In addition to supporting the primary study aim of evaluating the efficacy of robot-mediated therapy in reducing elbow spasticity, these measures will enable deeper insights into the complex interplay of neuro-physiological mechanisms underlying spasticity, hyper-resistivity, and functional recovery, to date insufficiently understood (14, 46).

This way, the proposed protocol fulfills all 4 pillars of Duret's framework for effective upper-limb hemiparesis rehabilitation via repetitive, intensive, adaptive, and quantifiable physiotherapy. While numerous investigations have employed robotic devices in upper limb stroke rehabilitation, there remains a lack of studies that have integrated all four of these aspects in specifically addressing upper limb spasticity. Rather, the majority of upper limb robotic rehab studies, including (12, 23, 41), have used robotic devices for the administration of therapy and/or for instrumented measurement of movement quality but have relied on clinical scales (typically the MAS) to assess limb spasticity and rigidity, which remain subject to the severe limitations in reliability, sensitivity (precision), and physiological specificity noted above.

By comparison, fewer studies have investigated the relationship between muscle activity and biomechanics in the spastic elbow. For instance, despite reporting significant improvements in MAS and FMA scores following 6 weeks of robot-assisted therapy in upper limb stroke patients, Frisoli et al. (12) found no significant correlation between these clinical measures and co-contractions of the *BB and TB* muscles during a separate kinematic-plus-EMG analysis of free reaching movements performed *outside* of the robot. Moreover, this separation of robot use and EMG analysis did not enable the direct relation of muscle activity to limb hyper-resistance, while their study excluded subjects with severe spasticity (MAS > 2).

Perhaps the most significant combinations of robotic and EMG recordings for spasticity assessment in stroke patients have been performed by the groups of Mullick (38) and Sin (22). The former found that neurally regulated spasticity could be distinguished from elbow stiffness via measurement of spatial stretch reflex thresholds (ST) induced by elbow F/E movements at different movement velocities, actuated by a custom-automated “manipulandum” device. However, these instrumented measures exhibited no correlation with the Composite Spasticity Index, a clinical spasticity measure which includes MAS. Meanwhile, Sin et al. developed a robotic-plus-EMG-instrumented spasticity assessment technique based on the angle of catch (measured using *either* EMG activity *or* joint torque) during isokinetic elbow F/E movements at 150°/s, which demonstrated excellent test-retest reliability and improved inter-rater reliability with respect to manually effected movements. However, this measure was not directly compared with any clinical spasticity scales. Critically, neither of these studies employed their instrumented spasticity measures in the context of rehabilitation program (robotic or otherwise), nor did they analyze synchronous EMG measurements and joint torques to elucidate the relationship between elbow spasticity and hyper-resistance.

In sum, the proposed RCT incorporates numerous previously disparate elements and benefits of upper limb robotic rehabilitation and spasticity assessment techniques into a single integrated investigation that embodies current clinical best-practice guidelines. It is important to highlight that the intent of this work is not to promote, diminish, or prefer one approach to the other, but rather to underline advantages and disadvantages in the adoption of both techniques, possibly enriching the clinical landscape with a new procedure in which one complements the other, compensating for the low granularity and repeatability exhibited by most of the commonly adopted clinical practices. In addition to a controlled clinical investigation of the efficacy of exoskeletal robotic therapy for post-stroke spasticity management, the proposed study protocol aims to validate a new instrumented spasticity assessment technique that may form the basis of a more reliable, sensitive, and standardized clinical spasticity measure in the future. Moreover, the extraction of advanced biomechanical and performance parameters from the NEEM system (PI, aROM, nTAI, etc.) during therapy sessions will provide additional “real-time” measures of patient recovery that may be further used by neurorehabilitation clinics in the future to functionally integrate patient assessment and treatment paradigms, as well as to dynamically adapt physiotherapy protocols in accordance with individual patients' capabilities and needs. In this way, the proposed clinical trial will provide a firm foundation for the continued optimization and personalization of technologically-enabled neuro-rehabilitative care.

## Data Availability Statement

The original contributions presented in the study are included in the article/supplementary material, further inquiries can be directed to the corresponding author/s.

## Ethics Statement

The studies involving human participants were reviewed and approved by Ethical Committee for the Azienda USL Toscana Nord Ovest (AUSLTNO) and Italian Ministry of Health. The patients/participants provided their written informed consent to participate in this study.

## Author Contributions

AP, ET, and ZM participated in the design of the study protocol and drafted the manuscript. CF and CM participated in the design of the study protocol. FP, SC, and NV conceived and supervised the study protocol and contributed to its design, revised and edited the manuscript. All authors approved the submitted version of the manuscript.

## Conflict of Interest

SC and NV have commercial interests in IUVO S.r.l., a spinoff company of Scuola Superiore Sant'Anna. Currently, part of the IP protecting the NEEM has been licensed to IUVO S.r.l. for commercial exploitation. The remaining authors declare that the research was conducted in the absence of any commercial or financial relationships that could be construed as a potential conflict of interest.

## Abbreviations

aROM: active Range Of Motion
BB: biceps brachii
BR: brachioradialis
CG: control group
F/E: flexion/extension
NEEM: NEUROExos Elbow Module
PI: Performance Index
pROM: passive Range of Motion
RAT: robotic assisted training
TB: triceps brachii
TG: treatment group.
